# MB4-2/MB4-3 transcripts of *IGH-MMSET* fusion gene in t(4;14)^pos^ multiple myeloma indicate poor prognosis

**DOI:** 10.18632/oncotarget.18209

**Published:** 2017-05-24

**Authors:** Feng Li, Yong-Ping Zhai, Ting Lai, Qian Zhao, Hui Zhang, Yu-Mei Tang, Jian Hou

**Affiliations:** ^1^ Myeloma and Lymphoma Center, Department of Hematology, Changzheng Hospital, The Second Military Medical University, Shanghai, China; ^2^ Department of Hematology, Jinling Hospital, School of Medicine, Nanjing University, Nanjing, China

**Keywords:** t(4;14) translocation, *MMSET* gene, major breakpoint (MB4), prognosis, bortezomib

## Abstract

Multiple myeloma (MM) patients with t(4;14) is a heterogeneous group. Prognostic tools capable of predicting the outcome of patients are currently lacking. The MM SET domain (MMSET) protein is universally overexpressed and has been suggested to have an important tumorigenic role. This study analyzed whether the overexpression of full-length (MB4-1) or truncated forms (MB4-2 and MB4-3) of *MMSET* influence the prognosis of t(4;14)^pos^ MM patients. A total of 53 symptomatic t(4;14)^pos^ MM patients were retrospectively analyzed. RT-PCR was performed using cDNA from purified CD138+ bone marrow plasma cells to analyze expression and clinical significance of the *IGH-MMSET* fusion transcripts corresponding to MB4-1, MB4-2 and MB4-3 breakpoints. Among the patients, 25 (47.2%), 12 (22.6%) and 16 (30.2%) had the MB4-1, MB4-2 and MB4-3 breakpoints, respectively. When adjusted to the established prognostic variables including del(17p), ISS stage, serum LDH and serum calcium levels, the pooled MB4-2/MB4-3 subgroup remained a powerful independent adverse factor for PFS (*P*=0.013) and OS (*P*=0.029). Bortezomib-based therapy significantly improved the survival of the MB4-1 subgroup but could not overcome the negative effect of the MB4-2/MB4-3 breakpoints. Our results indicate that MB4-2/MB4-3 breakpoints with truncated forms of MMSET define a subset of t(4;14)^pos^MM with poor prognosis.

## INTRODUCTION

Multiple myeloma (MM) is an incurable clonal plasma cell disorder. Previous molecular studies have demonstrated that the disease is associated with several chromosomal translocations [1]. The t(4;14) translocation is the second most common type of these translocations, affecting approximately 15% of MM patients with symptomatic disease [2]. Clinical studies showed that MM patients carrying the t(4;14) translocation were resistant to traditional chemotherapy, resulting in short median overall survival [3–7]. An Intergroupe Francophone du Myélome (IFM) 99 study reported that t(4;14) translocation was an independent prognostic factor for survival along with 17p deletion and high β2-microglobulin (β2-MG) [7]. Although recent therapeutic regimens such as bortezomib-based induction improve the outcome of patients with t(4;14), the prognosis is still poor [8, 9]. Studies also suggested that MM patients with t(4;14) may be a heterogeneous group with both “high risk” and “good risk” patients [10].

The t(4;14) translocations in MM divide the strong 3’alpha and mu enhancers of the IgH locus into different derivative chromosomes. These translocations result in the 3’ alpha enhancers expressing *fibroblast growth factor receptor 3* (*FGFR3*), and the mu enhancer increases the expression of *multiple myeloma SET domain*(*MMSET*) [11]. Thus, two potential oncogenes, *FGFR3* and *MMSET*, are dysregulated in patients with t(4;14) translocations. *FGFR3* expression, which is lost in a subset of t(4;14)^pos^ MM, has been shown not to have a significant impact on patients’ survival [11–14]. The over-expressed *MMSET* gene of all t(4;14)^pos^ MM patients encodes a histone methyltransferase that is involved in tumor progression and genomic instability [13, 15, 16]. MB4-1, MB4-2, and MB4-3 are three major breakpoints within the 5’coding region of *MMSET* at 4p16 on chromosome der(4) [11, 16]. Each breakpoint overexpresses a specific *IGH/MMSET* fusion transcript. The hybrid transcripts from MB4-1 patients encode the full-length MMSET protein, while hybrid transcripts from MB4-2 patients lack the first translated exon of *MMSET*. MB4-3 patients lack the first and second translated exons of *MMSET*. The aim of this study was to clarify whether the overexpression of full-length (MB4-1) or truncated forms (MB4-2 and MB4-3) of *MMSET* influences the prognosis of MM patients with t(4;14).

## RESULTS

### MB4 breakpoints distribution and *FGFR3* expression in t(4;14)^pos^ MM

**Figure 1:**
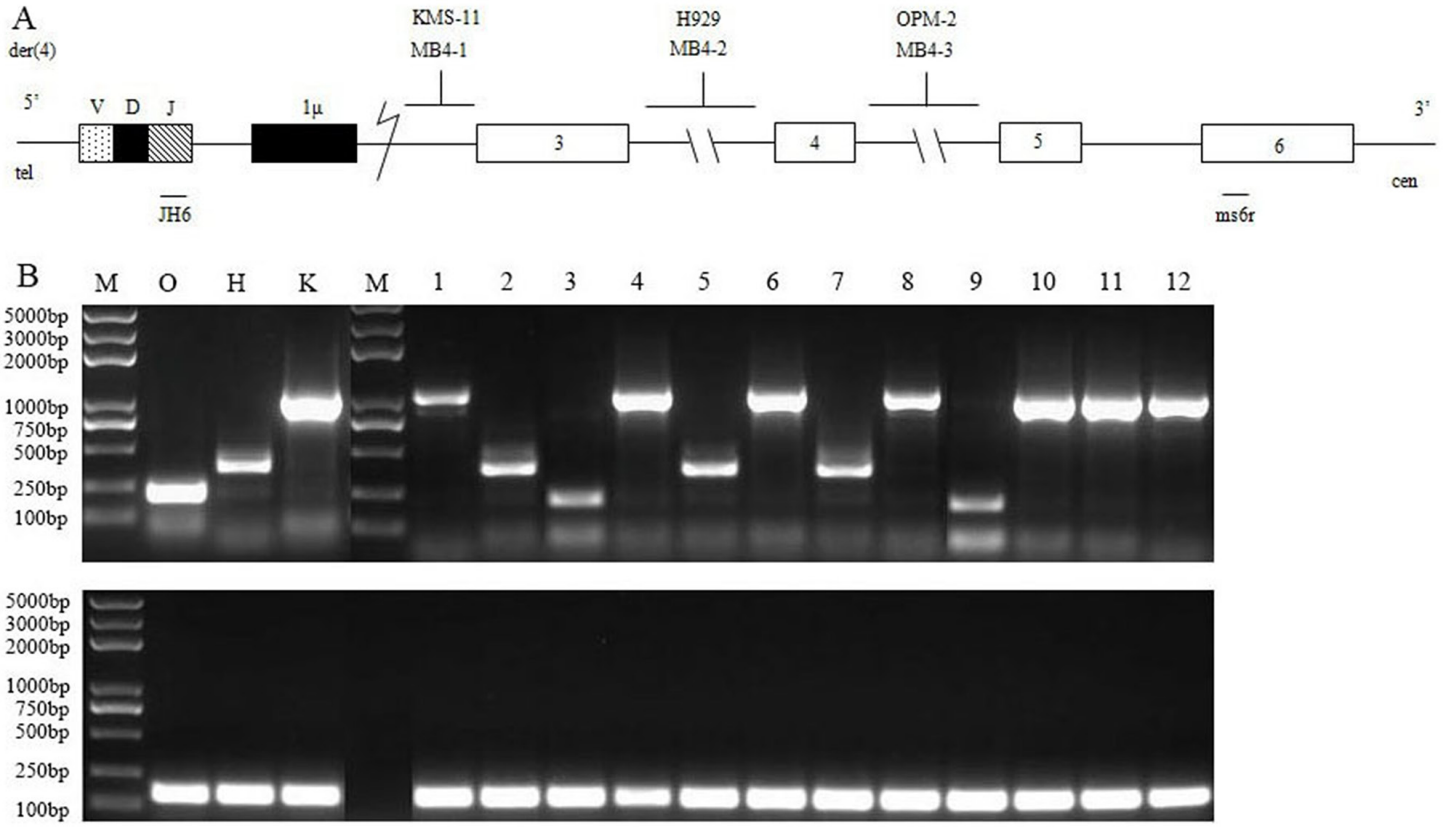
RT-PCR assay of detecting *IGH/MMSET* hybrid transcripts associated with the t(4;14)(p16.3;q32) translocation in MM **(A)** Schematic representation of the t(4;14) junction, der(4), showing the three different types of 4p16.3 breakpoints (see the text): the *MMSET* exons (□) and the *IGH* region (Iμ, ■; JH, ▧) were indicated. **(B)** RT-PCR analysis of the *IGH/MMSET* hybrid transcripts in the KMS-11 (K), NCI-H929 (H) and OPM-2 (O) cell lines and the patient samples using the JH6 and ms6r primers.

Fifty-three MM patients with t(4;14) were sub-grouped into 3 different major breakpoint regions (MB4-1, MB4-2 and MB4-3) based on the size of the RT-PCR products (Figure 1B). Of the 53 t(4;14)^pos^ patients, 25 (47.2%), 12(22.6%) and 16(30.2%) had the MB4-1, MB4-2 and MB4-3 breakpoint, respectively. Due to the imbalance in the numbers of the three subgroups, we grouped them into two groups, MB4-1 subgroup (n=25) and the pooled MB4-2/MB4-3 subgroup(n=28), according to their ability to encode a full-length or a truncated MMSET protein. *FGFR3* expression was detectable in 43(81.1%) of the 53 MM with t(4;14). For the subgroups, *FGFR3* expression was detected in 22(88%) of the MB4-1 subgroup and 21(75%) of the MB4-2/MB4-3 pooled subgroup, with no statistical difference between them (*P*=0.302).

### Patient characteristics

The median age of the 53 patients was 60 years old (range 42–85) with the median follow-up time of 18.83 months from the diagnosis. Clinical factors, genetic abnormalities and treatments associated with MB4 breakpoints are shown in Table 1. There was no significant difference in clinical and cytogenetic characteristics and in responses after induction therapy between the two groups.

**Table 1:** Characteristics of patients with symptomatic MM (N=53), grouped by MB4 breakpoints

Breakpoint	MB4-1	MB4-2/MB4-3	*P*
n (%)	25 (47.2)	28 (52.8)	
Gender (male, %)	17 (68)	17 (60.7)	0.775
Median age, years (range)	59 (42-85)	60 (49-74)	>0.05
M isotype,n (%)			0.305
IgA	11 (44)	7 (25)	
IgG	11 (44)	18 (64.3)	
IgD	0 (0)	1 (3.57)	
Light chains, n (%)	3 (12)	2 (7.14)	
BM plasmacytosis≥50%, n (%)	7	4	0.219
Albumin (g/L) ≤ 35, n (%)	13 (52)	17 (60.7)	0.586
Calcemia ≥2.8 mmol/L, n (%)	2 (8)	3 (10.7)	1
Creatinine≥176 μmol/L, n (%)	5 (20)	8 (28.6)	0.536
Elevated lactate dehydrogenase, n (%)	4 (16)	10 (35.7)	0.129
Involved/uninvolved serum free light ratio≥100, n (%)	6 (24)	9 (32.1)	0.556
Anemia (g/L), n (%)			0.969
>100	7 (28)	8 (28.6)	
80~100	7 (28)	7 (25)	
<80	11 (44)	13 (46.4)	
Number of osteolytic destruction (≥3), n (%)	19 (76)	18 (64.3)	0.387
Extramedullary invasion, n (%)	4 (16)	8 (28.6)	0.337
DS, n (%)			1
I+II	2 (8)	2 (7.1)	
III	23 (92)	26 (92.9)	
R-ISS, n (%)			0.237
I+II	20 (80)	18 (64.3)	
III	5 (20)	10 (35.7)	
*FGFR3* expression, n (%)	22 (88)	21 (75)	0.302
Cytogenetic abnormality			
Del(13q)	17 (68)	23 (82.1)	0.339
Del(17p)	5 (20)	6 (21.4)	1
Amp(1q21)	13 (52)	19 (67.9)	0.272
High-risk [(any del(17p) or amp(1q21)]	14 (56)	21 (75)	0.162
Induction treatment, n=53			
Bortezomib-based regimen, n (%)	19 (76)	16 (57.1)	0.148
Immunomodulatory drug-based regimen, n (%)	3 (12)	8 (28.6)	0.138
Traditional chemotherapy, n (%)	3 (12)	4 (14.3)	1
High dose therapy + ASCT, n (%)	4 (16)	5 (17.9)	1
Response after 4 cycles induction therapy, n=53			0.54
Partial response, n (%)	4 (16)	5 (17.9)	1
Very good partial response, n (%)	10 (40)	12 (42.9)	1
Complete response, n (%)	8 (32)	4 (14.3)	0.19
Stable disease, n (%)	2 (8)	3 (10.7)	1
Progressive disease, n (%)	1 (4)	4 (14.3)	0.355
Overall response, n (%)	22 (88)	21 (75)	0.302
CR after two cycles of induction therapy, n (%)	5 (20)	1 (3.6)	0.089
VGPR after two cycles of induction therapy, n (%)	11 (44)	11 (39.3)	0.785

R-ISS: Revised International Staging System; Ig: immunoglobulin; BM: bone marrow.

### Prognostic value of MB4 breakpoints in newly diagnosed MM patients with t(4;14)

**Figure 2:**
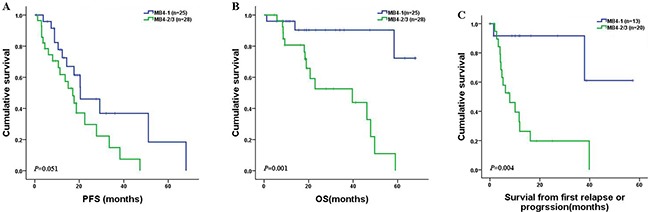
PFS, OS and survival in newly diagnosed MM patients with t(4;14) according to the MB4 breakpoints The patients with MB4-1 breakpoint had longer OS (*P*=0.001) **(B)** and survival **(C)** from the first relapse or progression (*P*=0.004) than those in the MB4-2/MB4-3 subgroup. However, the PFS (*P*=0.051) was similar between the two subgroups **(A)**.

The follow-up data of the 53 patients were analyzed to ascertain the prognostic value of MB4 breakpoints in newly diagnosed MM patients with t(4;14). We found that patients with MB4-1 breakpoint had similar progression free survival (PFS) to the patients in the pooled MB4-2/MB4-3 subgroup (20.5 vs. 17.1 months, *P*=0.051). However, the patients with MB4-1 breakpoint had significantly longer overall survival (OS) than the patients of the pooled MB4-2/MB4-3 subgroup (NS vs. 39.7 months, *P*=0.001) (Figure 2A and 2B). Accordingly, survival after the first relapse or progression was reduced in the pooled MB4-2/MB4-3 subgroup (median survival: NS vs 7.9 months, *P*=0.004) (Figure 2C). We further analyzed the remission rate after the first relapse or progression, and found that 9 out 13 (69.2%) patients in the MB4-1 subgroup and 6 out 20 (30%) patients in the MB4-2/MB4-3 subgroup achieved remission after the first relapse or progression. The remission rate difference was significant (*P*=0.038) with the MB4-1 subgroup superior to the MB4-2/MB4-3 subgroup. In addition, we also investigated the impact of *FGFR3* expression on PFS and OS of the 53 patients. No prognosis significance of the *FGFR3* expression was observed in the 53 patients with t(4;14) as a whole (data not shown). Furthermore, the expression of *FGFR3* had no prognosis significance in both MB4-1 and MB4-2/MB4-3 subgroups. We further analyzed other risk factors that might affect the prognosis of this cohort of patients (Table 2). The univariate analysis indicated that patients with ISS stage III, lactate dehydrogenase (LDH) higher than 245U/L, creatinine (Cr) higher than 176umol/L, ß2-MG higher than 5.5mg/L, del(17p), amp(1q21) and high-risk genetic abnormality had inferior OS to the corresponding control group. Furthermore, patients with serum calcium higher than 2.8mmol/L, del(17p) and high-risk cytogenetic abnormality had inferior PFS to the corresponding control group. Multivariate analysis of the above mentioned prognostic variables showed that t(4;14) grouped into MB4-1 and MB4-2/MB4-3 subgroups according to the breakpoints remained a powerful independent adverse factor for PFS (HR 2.74, 95% CI: 1.24–6.09, *P*=0.013) and OS (HR 4.37, 95% CI: 1.17–16.33, *P*=0.029). Other independent factors for OS were del(17p) (HR 3.65, 95% CI: 1.2–11.14, *P*=0.023), ISS stage III (HR 3.76, 95% CI: 1.19–11.9, *P*=0.024) and LDH (HR 2.86, 95% CI: 1–8.19, *P*=0.05). For PFS, serum calcium was another independent factor (HR 11.54, 95% CI: 3.7–36, *P*< 0.001) (Table 3).

**Table 2:** Univariate analysis of risk factors for PFS and OS in the 53 newly diagnosed MM patients with t(4;14)

Prognostic parameters	Median PFS (months)	*P* value	Median OS (months)	*P* value
ISS stage		0.344		0.001
I-II (n=38)	20.4		58.4	
III (n=15)	15.03		20.7	
LDH(U/L)		0.764		0.033
≥245 (n=15)	17.1		18.97	
<245 (n=38)	20.4		47.9	
ß2-MG(mg/L)		0.756		0.039
≥5.5 (n=16)	27.7		39.7	
<5.5 (n=37)	18.7		58.4	
Cr(μmol/L)		0.477		0.03
≥176 (n=13)	27.7		39.7	
<176 (n=40)	18.7		58.4	
Plasma counts in BM(%)		0.699		0.256
≥50 (n=11)	12.4		49.7	
<50 (n=42)	18.7		47.9	
Number of osteolytic destruction		0.609		0.563
≥3 (n=37)	18.7		47.87	
<3 (n=16)	38.27		59	
Extramedullary invasion		0.124		0.336
Positive (n=12)	11.33		20.7	
Negative (n=41)	20.4		58.4	
Serum calcium(mmol/L)		< 0.001		0.148
≥2.8 (n=5)	3.07		NS	
<2.8 (n=48)	20.5		49.67	
sFLC ratio (involved/uninvolved)		0.756		0.374
≥100 (n=15)	33.5		47.9	
<100 (n=38)	18.7		58.4	
T(4;14)/MB4 breakpoint		0.051		0.001
MB4-1 (n=25)	20.5		NS	
MB4-2/MB4-3 (n=28)	17.1		39.7	
Del(13q)		0.728		0.77
Positive (n=40)	20.4		47.9	
Negative (n=13)	15.03		NS	
Del(17p)		0.001		0.013
Positive (n=11)	10.6		20.7	
Negative (n=42)	22.5		58.4	
Amp(1q21)		0.144		0.028
Positive (n=32)	18.7		46.2	
Negative (n=21)	68		NS	
High-risk [(any del(17p) or Amp(1q21)]		0.028		0.003
Positive (n=35)	17.1		46	
Negative (n=18)	68		NS	

**Table 3:** Multivariate analysis of risk factors for PFS and OS in the 53 newly diagnosed MM patients with t(4;14)

Prognostic parameter	HR for PFS (95% CI)	*P* value	HR for OS (95% CI)	*P* value
MB4-2/MB4-3 (n=28)	2.74(1.24-6.09)	0.013	4.37(1.17-16.33)	0.029
ISS stage III (n=15)	-	0.717	3.76(1.19-11.9)	0.024
Del(17p) (n=11)	-	0.128	3.65(1.2-11.14)	0.023
LDH (n=15)	-	0.795	2.86(1-8.19)	0.05
Serum calcium (n=5)	11.54(3.7-36)	< 0.001	-	0.955

### Impact of *MMSET* breakpoints on the outcome of t(4;14)^pos^ patients and other cytogenetic high-risk factors

**Figure 3:**
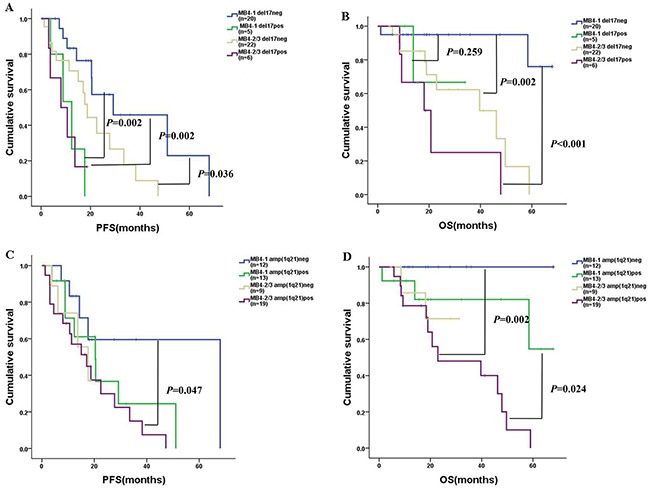
PFS and OS of the 53 patients according to the MB4 breakpoints and del(17p) /amp(1q21) **(A)-(B)**: MB4-1 without del(17p) (n=20): blue curve; MB4-1 with del(17p) (n=5): green curve; MB4-2/MB4-3 without del(17p) (n=22): brown curve; MB4-2/MB4-3 with del(17p) (n=6): purple curve. The MB4-1 without del(17p) subgroup had longer PFS than the other three subgroups (*P*=0.002, 0.036 and 0.002, respectively). MB4-1 without del(17p) subgroup had longer OS than MB4-2/MB4-3 with/without del(17p) (*P*<0.001 and *P*=0.002), however, the MB4-1 without del(17p) subgroup had similar OS compared to the MB4-1 with del(17p) subgroup (*P*=0.259). **(C)-(D)**: MB4-1 without amp(1q21) (n=12): blue curve; MB4-1 with amp(1q21) (n=13): green curve; MB4-2/MB4-3 without amp(1q21) (n=9): brown curve; MB4-2/MB4-3 with amp(1q21) (n=19): purple curve. The MB4-2/MB4-3 with amp(1q21) subgroup had shorter PFS than MB4-1 without amp(1q21) (*P*=0.047), but similar PFS compared to the other two subgroups (*P*=0.281 and 0.84). The MB4-2/MB4-3 with amp(1q21) subgroup had shorter OS than MB4-1 with or without amp(1q21) (*P*=0.024 and 0.002), but similar OS compared to the MB4-2/MB4-3 without amp(1q21) subgroup (*P*=0.61).

Deletion of chromosome 17 at p13 [del(17p)] and amplification of chromosome 1 at q21[amp(1q21)], which are associated with high-risk MM, were analyzed by fluorescence in situ hybridization(FISH) at diagnosis of the 53 cases. Overall, del(17p) was detected in 11 cases (20.8%). Amp(1q21) was found in 32 cases(60.4%). First, we analyzed PFS and OS of the 53 patients according to the MB4 breakpoints and del(17p). Interestingly, MB4-1 without del(17p) subgroup had longer PFS than the other three subgroups (*P*=0.002, 0.036, 0.002, respectively) (Figure 3A). MB4-1 without del(17p) subgroup had longer OS than MB4-2/MB4-3 with/without del(17p) (*P*<0.001 and *P*=0.002, respectively). However, MB4-1 without del(17p) subgroup had similar OS to MB4-1 with del(17p) subgroup (*P*=0.259) (Figure 3B). We also analyzed the PFS and OS of the 53 patients according to the MB4 breakpoints and amp(1q21). As shown in Figure 3C, MB4-2/MB4-3 with amp(1q21) subgroup had shorter PFS than MB4-1 without amp(1q21) (*P*=0.047), but similar to the other two subgroups (*P*=0.281 and 0.84, respectively). MB4-2/MB4-3 with amp(1q21) subgroup had shorter OS than MB4-1 with or without amp(1q21) (*P*=0.024 and 0.002, respectively), but similar to MB4-2/MB4-3 without amp(1q21) subgroup (*P*=0.61) (Figure 3D). MB4-1 without del(17p) subgroup may be a subset of t(4;14)^pos^ MM with superior prognosis. However, one should note that these comparisons have small sample sizes.

### Bortezomib significantly improved the survival of MB4-1 patients

**Figure 4:**
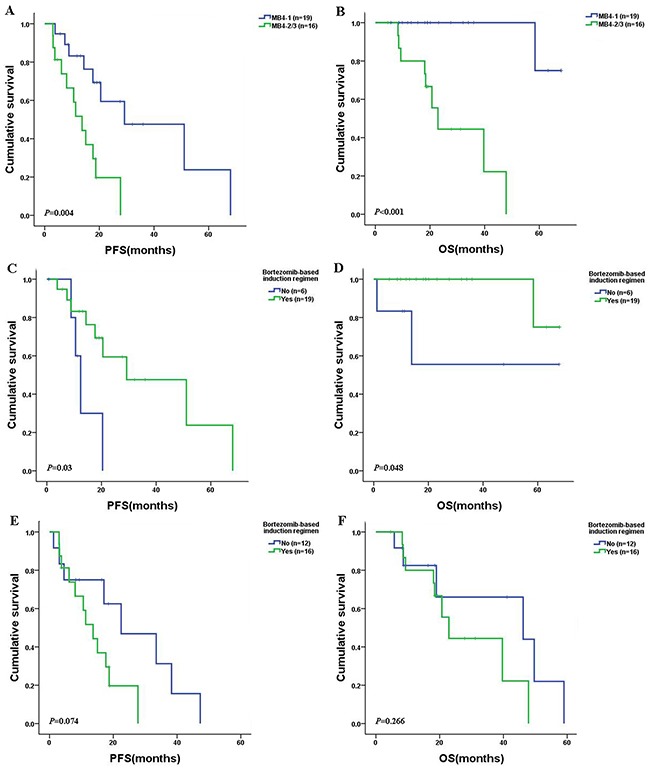
PFS and OS in newly diagnosed MM patients with t(4;14) after receiving bortezomib-based or other chemotherapies **(A)-(B)**: The MB4-1 patients had longer PFS (*P*=0.004) and OS (*P*< 0.001) than that of MB4-2/MB4-3 subgroup. **(C)-(D)**: The MB4-1 patients treated with bortezomib-based chemotherapy had longer PFS (*P*=0.03) and OS (*P*=0.048) than those treated with other chemotherapies. **(E)-(F)**: The MB4-2/MB4-3 patients had similar PFS and OS between bortezomib-based and other chemotherapies groups (PFS: *P*=0.074; OS: *P*=0.266).

In this study, the majority of the patients received a bortezomib-based induction regimen (Table 1). The overall response rate (ORR) of the patients in the MB4-1 subgroup was superior to that in the MB4-2/MB4-3 subgroup after being treated with the bortezomib-based induction therapy (*P*=0.035) (Table 4). In the bortezomib-based chemotherapy group, the median PFS and OS of patients with MB4-2/MB4-3 breakpoints were 13.67 vs. 29.2months (*P*=0.004) and 22.9 vs. NS (*P*=0.00), respectively, when compared to the patients with MB4-1 breakpoint (Figure 4A and 4B). In addition, in the patients with MB4-1 breakpoint, the median PFS and OS of the patients treated with bortezomib-based chemotherapy were 29.2 vs. 12.37months (*P*=0.03) and not reached vs. 41.73 months (*P*=0.048), respectively, when compared to the patients treated with other chemotherapies (Figure 4C and 4D). However, for patients with the MB4-2/MB4-3 breakpoints, no statistically significant difference between bortezomib-based and other chemotherapy groups was observed (PFS: *P*=0.074; OS: *P*=0.266) (Figure 4E and 4F). This suggested that bortezomib significantly improved the survival of the patients with MB4-1 but could not overcome the negative effect of the MB4-2/MB4-3 breakpoints.

**Table 4:** Response rate of patients with different MB4 breakpoints treated with bortezomib-based induction therapy

Breakpoint	ORR	PR	VGPR	CR	SD	PD	≥VGPR	SD+PD
MB4-1 (n=19)	100% (19/19)	15.8% (3/19)	47.4% (9/19)	36.8% (7/19)	0 (0/19)	0 (0/19)	84.2% (16/19)	0 (0/19)
MB4-2/MB4-3 (n=16)	75% (12/16)	18.8% (3/16)	37.5% (6/16)	18.8% (3/16)	6.3% (1/16)	18.8% (3/16)	56.3% (9/16)	25% (4/16)
*P* value	0.035	1	0.734	0.285	0.457	0.086	0.132	0.035

## DISCUSSION

T(4;14) translocation affects approximately 15% of MM patients with symptomatic disease. The 53 patients in this retrospective study were identified from nearly 400 symptomatic MM patients diagnosed between June 2011 and August 2016 in two hospitals in China. Our study revealed that *FGFR3* expression was detectable in 81.1% of the 53 MM patients with t(4;14). No prognostic significance of *FGFR3* expression was observed in the present study. This finding is consistent with previous reports [16, 17]. We found that of the 53 t(4;14)^pos^ MM patients, 47.2% had the breakpoint of MB4-1, while 22.6% and 30.2% of them had the MB4-2 and MB4-3 breakpoints, respectively. These results were different from those of a previous study by Lazareth et al. (2015), who found that MB4-1, MB4-2 and MB4-3 transcripts were expressed in 62%, 21% and 17% of their 256 symptomatic MM patients in France [17]. There was no doubt that the MB4-1 breakpoint cluster was the most common type of t(4;14)^pos^ MM patients. The distribution difference of the three MB4 breakpoints among patients in different studies might be due to the smaller sample size of the current study and/or different genetic background (e.g. race) of the studied patients.

In the current study, we found that the patients of MB4-1 and MB4-2/MB4-3 subgroups had a similar PFS but the post relapse survival of the later subgroup was shorter leading to a shorter OS. This result agrees with Lazareth et al. [17] where they found the similar result that MB4-2 was an independent prognostic factor for OS. The remission rate after the first relapse or progression of the MB4-1 subgroup was better than that of the MB4-2/MB4-3 subgroup in the current study. We supposed that symptomatic t(4;14)^pos^ MM patients with the MB4-2/MB4-3 breakpoints might be as sensitive as those with the MB4-1 breakpoint to the first-line therapy, but developed chemo-resistant relapse quickly resulting in poorer outcome. Lazareth et al. (2015) found that patients with del(17p) and the MB4-2 breakpoint formed a distinctive subset of high risk patients with the very poor prognosis [17]. However, Keats et al. (2005) found similar OS between MB4-1 (n=30) and MB4-2/MB4-3 (n=13) subgroups with a small number of patients [16]. Our current study also had a limited number of cases (53). We failed to further distinguish the prognosis between MB4-2 and MB4-3 subgroups.

For the first time, we verified that bortezomib-based therapy significantly improved the survival of patients with MB4-1, but could not overcome the negative effect of the MB4-2/MB4-3 breakpoints, with unclear mechanism. The study by Chng WJ et al. [18] indicated an interaction between MMSET and the nuclear factor-κB and they both bound to the *interferon regulatory factor 4 (IRF4)* promoter region which is critical for MM cell survival. Furthermore, they found that bortezomib could reduce the expression of *MMSET* and *IRF4*. This might be one reason that bortezomib-based therapy significantly improved the survival of patients with full-length MMSET (MB4-1 subgroup) rather than that of truncated forms (MB4-2 and MB4-3 subgroup). Further studies are needed to clarify the difference between MB4-1 and MB4-2/MB4-3's responses to bortezomib and survival. Currently, all the results suggested that the MB4-2/MB4-3 subgroup was at “high risk”, while MB4-1 subgroup was at “good risk” in MM patients with t(4;14). In our study, all the patients with del(17p) using 20% as cut-off level had more than 50% of 17p deletion cells among clonal plasma cells [19]. We did not find MB4-2/MB4-3 patients with del(17p) or amp(1q21) having higher risk with very poor prognosis, probably due to the limited number of cases.

The molecular basis for the particularly poor prognosis associated with the MB4-2 and MB4-3 breakpoints is not clear. Knockdown studies have demonstrated that *MMSET* upregulation contributes to cellular adhesion, clonogenic growth and tumorigenicity [13–16, 20, 21]. However, *MMSET* overexpression is not the only factor as t(4;14)^pos^ patients have genomic breakpoints that separate the first, or first and second, translated exons from the remaining translated exons. Keats et al. (2005) [16] showed that the full-length MMSET proteins (MB4-1) concentrated at the nucleus, whereas the MB4-2 and MB4-3 proteins concentrated in nucleoli. The domain for controlling MMSET localization exists in the N-terminus encoded by exons 3 and 4, which are lost in the MB4-2 and MB4-3 variants. Cloning and localization studies of the Exon 4a/MMSET III splice variant (absent from the MB4-2 variant) identified a novel protein domain that prevented nucleolar localization. Protein location difference may cause functional divergence. MB4-1 expressed MMSET proteins have a complete structure with the N-terminal region that limits the protein entering of cell nucleus. The MB4-2 and MB4-3 breakpoints truncate the N-terminal region, and thus lack the control of MMSET proteins entering the nucleus. Therefore, it is reasonable to assume that the poor prognosis of MM patients with MB4-2 and MB4-3 breakpoints is due to the accumulation of fusion proteins in the cell nucleus with stronger cancer causing force. More recently, Debra L. Evans [22] reported that during cell-cycle progression, MMSET interacted with proliferating cell nuclear antigen (PCNA, a sliding clamp for DNA synthesis) through its N terminus and was degraded during synthesis (S) phase in a PCNA-dependent manner. N terminus of MMSET was absent in MB4-2 and MB4-3 breakpoints. This would be one reason for the poor prognosis of MM patients with MB4-2 and MB4-3 breakpoints.

MMSET has also been implicated in the H4K20 histone methyltransferase activity associated with the cellular response to DNA damage [15]. This function takes effect through the phosphorylation of serine 102 (Ser102) by the ATM protein, which promotes the binding of MMSET at DNA double-strand breaks and recruits p53-binding protein 1 (53BP1) [15]. 53BP1 is known to be an important mediator of the DNA damage response [15]. Therefore, the absence of Ser102 in truncated MMSET isoforms might result in genomic instability and the emergence of resistant clones [17]. These could likely account for the different clinical outcomes observed for MB4-1 and MB4-2/MB4-3 breakpoints. Future studies will be needed to explain the apparent discrepancy in prognosis of the MB4-1 and MB4-2/MB4-3 breakpoints.

In conclusion, our results indicated that patients with the MB4-2/MB4-3 breakpoints had more adverse prognosis and higher resistance to bortezomib-based therapy compared to those with the MB4-1 breakpoint. Thus, the breakpoints on the *MMSET* locus may partially explain the prognostic heterogeneity of t(4;14)^pos^ MM with unclear mechanism. Based on the results, a systemic identification of the MB4 breakpoints may be useful in the management of MM patients with t(4;14) for more accurate therapy and improved outcome.

## MATERIALS AND METHODS

### Patients and samples

The study involved 53 newly diagnosed symptomatic MM patients at Changzheng Hospital of Shanghai or Jinling Hospital of Nanjing, China, between June 2011 and August 2016. All patients were detected as t(4;14) positive by FISH at diagnosis. Bone marrow (BM) aspirates were obtained from these patients after informed consent. Mononuclear cells were separated from patients’ BM by gradient density centrifugation (Ficoll-Hypaque; Eurobio, Les Ulis, France). Plasma cells were then purified using CD138-coated magnetic beads according to the manufacturer's instructions (Miltenyi Biotec, Germany) to ensure greater than 90% plasma cell purity. At least 1×10^6^ plasma cells were frozen in Trizol at -80°C for the extraction of mRNA. The remaining CD138+ plasma cells were detected by FISH. This study was approved by the Ethics Committee of Changzheng Hospital.

The last follow-up date was December 31, 2016. The median follow-up time was 18.83 months from the diagnosis. Median cycles of induction and consolidation chemotherapy were 6 and 8 for MB4-1 subgroup and MB4-2/MB4-3 subgroup, respectively. Nineteen MB4-1 patients received at least 4 cycles of bortezomib-based induction therapy with nine cases of CBD (bortezomib, cyclophosphamide and dexamethasone), one case of VD (bortezomib and dexamethasone), eight cases of PAD (bortezomib, adriamycin and dexamethasone), and one case of VTD (bortezomib, thalidomide and dexamethasone). Three MB4-1 patients received at least 4 cycles of the induction therapy of immunomodulatory drug-based regimen with one case of RCD (lenalidomide, cyclophosphamide and dexamethasone), and two cases of CTD (cyclophosphamide, thalidomide and dexamethasone). The remaining three MB4-1 patients received at least 4 cycles of the traditional VAD chemotherapy (vindesine, doxorubicin or pegylated doxorubicin and dexamethasone). Sixteen MB4-2/MB4-3 patients received at least 4 cycles of bortezomib-based induction therapy with six cases of CBD, one case of VD, eight cases of PAD and one case of VTD. Eight MB4-2/MB4-3 patients received at least 4 cycles of immunomodulatory drug-based induction therapies with three cases of TAD (thalidomide, doxorubicin and dexamethasone), two cases of BiCTD (clarithromycin plus CTD), one case of CTD, one case of RD (lenalidomide and dexamethasone) and one case of RAD (lenalidomide, doxorubicin and dexamethasone). The remaining four MB4-2/MB4-3 patients received at least 4 cycles of VAD.

The treatment responses were evaluated according to the IMWG criteria [23] for complete response (CR), very good partial response (VGPR), partial response (PR) and stable disease (SD). Treatment effect was evaluated by ORR, which is the combination of CR, PR and VGPR. OS and PFS were also defined according to the IMWG criteria. OS is defined as the time from diagnosis to death. PFS is defined as the duration from treatment commencement to disease progression or death (regardless of the cause of death), whichever comes first.

### Cell lines

Three cell lines, MM-derived KMS-11, NCI-H929 and OPM-2, represent the following three 4p16.3 breakpoint patterns of the *MMSET* gene described thus far: (a) 5’to exon 3 (KMS-11); (b) within intron3 (NCI-H929); or (c) within intron 4 (OPM-2) (see the scheme in Figure 1A). For simplicity, MB4-1, MB4-2 and MB4-3 were used for these breakpoints, respectively. The cell lines were kindly provided to us by Dr. XinLiang Mao, Soochow University, Suzhou, China.

### Fluorescence in situ hybridization

The purified CD138+ plasma cells were assessed using DNA probes specific for the following chromosomal aberrations: del(13q14), del(17p) and amp(1q21). The probes were purchased commercially (Beijing Jinpujia Medical Treatment Science Co. Lt.). For each probe, 200 plasma cells were scored and the cut-off level was at 20% for both deletion and amplification according to the recommendation of the European Myeloma Network (EMN) [24]. An epifluorescence microscope equipped with CCD camera and appropriate filters was used to capture fluorescent images.

### RNA extraction and RT-PCR analysis

The mRNAs from KMS-11, OPM-2 and NCI-H929 cell lines and the purified CD138+ BM plasma cells of the studied patients were extracted using the Trizol reagent (Life Technologies, Inc., Grand Island, NY). First-strand cDNA was synthesized using the PrimeScript™ 1^st^Strand cDNA Synthesis Kit (TaKaRa, Dalian, China). The PCR amplification reactions consisted of 5ml of the first-strand cDNA from each case mixed with 25ml PCR mixture containing specific primers (20pmol/L), MgCl_2_ (1mM), deoxynucleotide triphosphates (200mM) and Taq DNA polymerase (TaKaRa, Dalian, China) as described previously [25]. The primers for *IgH/MMSET* were as follows: JH6, ACCACGGTCACCGTCTCCTCA (sense primer); ms6r, CCTCAATTTCCCTGAAATTGGTT (antisense primer). The primers for *FGFR3* used in the study were as follows: FGFR3-F, GCGGGCAATTCTATTGGGT (sense primer) and FGFR3-R, GGGAGATCTTGTGCACGGTG (antisense primer). The primers for ß-actin, a housekeeping gene, were as follows: ß-actin-F, TTAGCTGTGCTCGCGCTACTCTCTC (sense primer); and ß-actin-R, GTCGGATTGATGAAACCCAGACACA (antisense primer). Thirty-five amplification cycles were performed at 94°C for 30 s, 55°C for 30 s and 72°C for 1 min.

### Statistical analysis

SPSS version 20.0 software was used for statistical analysis of the data. Categorical variable comparisons were performed using Fisher's exact test or chi-square and non-parametric tests. Survival curves were obtained using the Kaplan-Meier method and significant differences between the curves were tested using the log-rank test. Multivariate analysis of the Cox Proportional-Hazard model was performed to identify variables associated with PFS and OS. A statistically significant difference was considered at *P*≤0.05.

## Abbreviations

MM: multiple myeloma
MMSET: multiple myeloma SET domain
FGFR3: fibroblast growth factor receptor 3
FISH: fluorescence in situ hybridization
RT-PCR: reverse transcription polymerase chain reaction
PFS: progression free survival
OS: overall survival
ISS: International Staging System
R-ISS: Revised International Staging System
LDH: lactate dehydrogenase
β2-MG: β2-microglobulin
Cr: creatinine
del(17p): deletion of chromosome 17 at p13
amp(1q21): amplification of chromosome 1 at q21
pos: positive
der: derivation
Ser: Serine
BM: bone marrow
CBD: bortezomib, cyclophosphamide and dexamethasone
VD: bortezomib and dexamethasone
PAD: bortezomib, adriamycin and dexamethasone
VTD: bortezomib, thalidomide and dexamethasone
RCD: lenalidomide, cyclophosphamide and dexamethasone
CTD: cyclophosphamide, thalidomide and dexamethasone
VAD: vindesine, doxorubicin or pegylated doxorubicin and dexamethasone
TAD: thalidomide, doxorubicin and dexamethasone
BiCTD: clarithromycin plus CTD
RD: lenalidomide and dexamethasone
RAD: lenalidomide, doxorubicin and dexamethasone
ORR: overall response rate
CR: complete response
VGPR: very good partial response
PR: partial response
SD: stable disease
IMWG: International Myeloma Working Group
EMN: European Myeloma Network.
